# The Treatment of Anemia by Hydrotherapy—with Special Reference to the Anemia of Hook-Worm Disease

**Published:** 1911-01

**Authors:** William Lee Secor

**Affiliations:** Kerville, Texas, and Chicago; Head of the Department of Physiology and Professor of Therapeutics in the Chicago College of Medicine and Surgery


### THE TREATMENT OF ANEMIA BY HYDROTHERAPY—WITH SPECIAL REFERENCE TO THE ANEMIA OF HOOK-WORM DISEASE.

By William Lee Secor, Ph.D., M. D., Kerville, Texas, and Chicago.

*Head of the Department of Physiology and Professor of Therapeutics in the Chicago College of Medicine and Surgery.*

In many parts of the South we see a great deal of anemia. Much of it is due either to hook-worm disease or to malaria, but many cases are seen in which evidence of neither of these diseases can be found. As these idiopathic cases seem to increase as we approach or pass the frost line in going South, it seems probable that the lack of the tonic effect of cold weather may be a causative factor.

The treatment generally applied in these cases of anemia and in chlorosis is arsenic or iron, depending on whether it is desired to increase the number of red cells in the blood or to raise the percentage of hemoglobin. Frequently a combination of these two drugs is used with good results.

The patient with pronounced hook-work infection is always anemic and after the intestine has been cleared of the parasite the principal treatment should be directed toward the anemia. In a large percentage of cases the system responds readily to the arsenic and iron medication and in children we see some very rapid and marked results from the use of the syrup of the iodide of iron. It is true, however, that there are some cases, in fact, quite a few, that do not respond readily to this line of treatment and after it has been tried for weeks or even months seem to show no improvement.

In all classes of simple anemia and chlorosis hydrotherapy has been proven of the very greatest value and frequently when drug medication has utterly failed to produce the desired result, a course of hydriatic applications will very soon demonstrate the positive action of this method of treatment.

I have time and again demonstrated to my satisfaction, by blood count and hemoglobin estimated that even a single hydriatic treatment will cause both an increase in the cellular elements of the blood and in the percentage of hemoglobin.

I do not believe that the very marked increase in blood cells that we often see after such hydriatic procedures, as the cold percussion douche, is due entirely to the manufacture of new cells, but it is produced by a throwing into the blood stream of latent corpuscles through the influence of the reflex effect upon the visceral circulation of stimulating applications to various skin areas that are in close connection with the viscera.

While a single application does not, to any great extent, actually increase the number of cells, nevertheless, the continued use of proper hydriatic procedures will usually show a positive increase in both cellular elements and percentage of hemoglobin.

The cold percussion douche is probably the most powerful hydriatic procedure at our disposal for the treatment of anemia. It does not follow, however, that most excellent results cannot be obtained by other measures of the greatest simplicity. An essential element in the treatment seems to be the production of some degree of shock to the periferal nerves which probably act reflexly upon the blood-making organs, stimulating them to increased activity.

The amount of shock advisable will depend upon the temperament and condition of the patient. Some highly sensitive patients of nervous temperament would be thrown into a state of collapse by a pail pour at seventy degrees, while others require a low temperature by percussion to produce the desired amount of shock.

With patients that are very sensitive to cold applications and those in a feeble condition very mild measures should be used at first and gradually increased in severity. The "wet hand rub" is a good measure to start with, or the "cold mitten friction" may be used. After a few aplications of this type more vigorous measures may be tried, as the dripping sheet rub or the pail pour and in institutions, the cold percussion douche.

An excellent measure to prescribe in connection with the proper drug medication is the morning cold plunge. The bath tub should be filled the night before so that the water will be about the temperature of the air then the patients should enter the plunge as soon as they get out of bed and not stand around to get the skin cooled off first. A single dip is all that is necessary, and three seconds in the water is better than a minute. Brisk friction with a Turkish towel should follow the bath and if the patient shows a good reaction the treatment has not been too severe. Those who are not vigorous enough for this procedure may use the wet hand rub each morning with much benefit.

If in the management of our hook-work cases, after we have used the regular thymol treatment, we employ a proper combination of drugs and hydrotherapy, we will get more rapid and satisfactory results. Extensive equipment is not necessary and good results can be obtained with the simplest measures.

